# Biofortification of Acacia and Polyflower Honey with *Pine sylvestris* L. Bud Extracts: Exploring Antioxidant Variation Across Developmental Stages for Enhanced Nutritional Value

**DOI:** 10.1007/s11130-024-01282-8

**Published:** 2025-01-25

**Authors:** Lidia Gizella Szanto, Romina Alina Marc, Andruța Elena Mureşan, Crina Carmen Mureșan, Andreea Puşacş, Floricuța Ranga, Florinela Fetea, Paula Ioana Moraru, Miuța Filip, Sevastița Muste

**Affiliations:** 1 Food Engineering Department, Faculty of Food Science and Technology, University of Agricultural Sciences and Veterinary Medicine, Cluj-Napoca, 400372 Romania; 2 Department of Food Science, Faculty of Food Science and Technology, University of Agricultural Sciences and Veterinary Medicine Cluj-Napoca, Cluj-Napoca, 400372 Romania; 3 Faculty of Agriculture, University of Agricultural Sciences and Veterinary Medicine Cluj- Napoca, Cluj-Napoca, 400372 Romania; 4 Raluca Ripan Institute for Research in Chemistry, Babeș-Bolyai University, 30 Fântânele Street, Cluj-Napoca, 400294 Romania

**Keywords:** Antioxidant properties, Honey products, Pine bud extract, Bioactive compounds, FTIR spectroscopy, DPPH radical scavenging

## Abstract

Honey is a valuable natural product with antioxidant properties, and its quality is influenced by various factors, including botanical origin and biofortification. Pine bud extracts, known for their antioxidant capacity, were explored to enhance the properties of acacia and polyflower honey. This study aimed to investigate the effect of pine bud extracts at different maturation stages on the moisture content, dry matter, antioxidant activity, and total phenolic content (TPC) of acacia and polyflower honey. Acacia and polyflower honey were biofortified with pine bud extracts at three maturation stages (Stage I, Stage II, and Stage III). Various analyses were performed, including moisture and dry matter content determination, total phenolic content measurement using the Folin-Ciocalteu method, and antioxidant activity assessment through DPPH radical scavenging. FTIR analysis was used to study the chemical composition of the biofortified honeys. Results showed that Acacia and polyflower honey maintained moisture content below 20%, with biofortification significantly enhancing their antioxidant profiles. The highest total phenolic content (247 ± 0.04 mg GAE/100 g) and DPPH scavenging activity (55 ± 0.05%) were observed in Acacia honey biofortified at maturation stage II. Polyflower honey exhibited increased phenolic content (232.9 ± 0.9 mg GAE/100 g) and antioxidant activity (52.45%) when biofortified at maturation stage II. Significant color changes were observed in polyflower honey, with a* and b* values increasing, indicating darker coloration. The biofortification of honey with pine bud extracts enhances its antioxidant and nutritional profile. This approach holds potential for the production of functional foods with improved health benefits. Further studies should explore its commercial feasibility and other potential bioactive compounds.

## Introduction

Functional foods provide essential nutrients and therapeutic effects. They offers various health benefits through bioactive ingredients with specific biological properties. Despite rapid growth, the functional food sector lacks regulation [1, 2]. Honey, a natural sweet food produced by bees (*Apis mellifera* L.) from floral nectar or plant secretions, is recognized for its functional properties promoting a healthy lifestyle, supported by research and clinical trials [3]. The global honey market was valued at over USD 7.5 billion in 2018, and it is projected to reach USD 10.5 billion by 2025. After China (27%), the European Union accounts for 13% of global honey output. In 2020–2021, Romania was the second-largest honey producer in Europe with 2,353,000 hives, following Spain (2,953,000 hives), and ahead of Poland and Switzerland [4].

The viscosity and sweetness of honey are attributed to the presence of fructose and glucose, with their ratio significantly impacting taste and consistency. The mineral contents such as calcium, iron, and potassium, as well as enzymes, can influence the color, flavor, and aroma of honey. The quantity and type of amino acids depend on the flower from which the bees collect nectar, but their influence on honey characteristics is minimal [5]. The Maillard reaction can occur during honey storage due to a decrease in the concentration of monosaccharides and an increase in the quantities of organic acids HMF (5-hydroxymethylfurfural and furosine). The Maillard reaction in honey occurs when amino acids and reducing sugars react, leading to browning and flavor changes. Honey’s low water activity (0.5–0.6) typically limits this reaction. However, increased moisture during storage can promote the Maillard reaction, with the breakdown of monosaccharides and formation of compounds like HMF facilitating this process [5, 6].

Food adulteration changes the composition of food, leading to a higher chance of poor-quality products. It involves intentionally reducing the quality of food by adding substandard ingredients, substituting key components, or removing essential elements. Adulteration of honey can occur during production through the use of various substances like glucose solutions, sucrose syrups, corn syrup, high fructose corn syrup, inverted sugar syrup, and sugar cane juice. Additionally, honey can be contaminated by environmental pollutants such as metal traces, polychlorobiphenyls, and pesticides from crops, as well as improper beekeeping practices involving medications and insecticides [7–9]. Consuming adulterated honey can be harmful to health as it may contain contaminants like dangerous trace elements that can affect its safety and quality [10, 11]. The increasing awareness of the benefits of healthy nutrition has driven a rise in fruit and fruit-based product consumption globally. In Poland, there is a variety of unique items available, including creamed honey with added dried herbs that may not have been extensively studied [12].

*Pinus sylvestris* L. contains antioxidants and is used in traditional medicine for its aromatic and medicinal properties. Pine shoots are a rich source of volatile compounds (beta-phellandrene, alpha and beta-pinene and ascorbic acid), polyphenolic compounds (caffeic acid, ferulic acid, and chlorogenic acid) which contribute to their antioxidant and pharmacological benefits, including expectorant and antispasmodic effects. The study by Grabek-Lejko et al. [13] found that honey fortified with Rubus components demonstrated antibacterial activity against *S. aureus.* While previous studies have highlighted the health benefits of honey, including its antioxidant properties, few have explored the effect of biofortification with plant extracts at different maturation stages. In spite of honey’s well-documented nutritional and medicinal properties, limited research has explored its improvement through biofortification with plant-derived extracts, particularly pine bud extracts at different maturation stages. This study addresses this gap by investigating how such biofortification influences key honey parameters, including moisture content, antioxidant activity, total phenolic content, and color. The novelty lies in establishing stage-specific correlations between biofortification and enhanced honey quality, providing insights into optimizing functional food products. This work has significant industrial relevance, offering a pathway for creating high-value, health-promoting honey varieties tailored to consumer needs.

## Materials and Methods

### Chemicals and Reagents

All reagents and chemicals used in this study were of analytical grade. Gallic acid, chlorogenic acid, and rutin which were used as standards for the HPLC-DAD-ESI-MS analysis, were purchased from Sigma-Aldrich (Steinheim, Germany). Folin–Ciocalteu’s phenol reagent, sodium carbonate (Na_2_CO_3_), sodium hydroxide (NaOH), acetic acid, acetonitrile, DPPH (2,2-diphenyl-1-picrylhydrazyl), and Trolox (6-hydroxy-2,5,7,8-tetramethylchroman-2-carboxylic acid) were also purchased from Sigma-Aldrich (Steinheim, Germany).

### Preparation of Pine Shoot Extract and Biofortification of Honey

Pine buds used in this study were collected from (mention place), The buds were identified botanically, authenticated by expert botanist. These were (mention whether it is wild or cultured), and their selection was based on maturity stages—Stage I (immature buds, light green and tender), Stage II (intermediate buds, yellow-green and semi-firm), and Stage III (mature buds, dark green to brown, fully firm). The pine buds harvested at three growth stages were dried. 5 g of each powder was added to 50 mL of methanol (99.9%) and stirred continuously for 120 h on a magnetic stirrer at 200 rpm. The solution was then filtered using Whatman filter paper no. 42. The filtrate was concentrated using a rotary evaporator, and the crude extract was stored for further analysis. The pine bud extracts from the three growth stages were combined with two types of honey (polyfloral and acacia) for 14 days. Following the maturation stages, six types of food supplements were obtained [14].

The honey extracts were obtained by mixing 10 g of honey with 25 mL of 99.9% methanol. The mixture was then centrifuged at 3000 rpm at 25 ºC for 10 min. The resulting supernatant was concentrated for 8 h to remove all methanol residues. The solution was then lyophilized overnight [15]. Methanol was chosen for extraction due to its superior efficiency in extracting phenolic compounds and antioxidants, which was critical for ensuring comprehensive bioactive compound recovery.

### Physicochemical Properties of Pine Shoot Extract and Pine Shoot- Fortified Honey

#### Determination of Total Acidity

The determination of total acidity involved titration with a standard volumetric solution of 0.1 N NaOH in the presence of phenolphthalein as an indicator. Pine bud samples were shredded using a Philips HR1614/00 650 W vertical blender. Extractions were carried out following the international standard (ISO 750:1998) (Fruit and Vegetable Products-Determination of Titratable Acidity, 1998) with some modifications. 5 g sample of pine buds was homogenized with 25 mL of distilled water to obtain a puree, which was then quantitatively transferred to a 50 mL volumetric flask. After centrifugation and filtration, 25 mL of the filtrate was titrated with 0.1 N NaOH solution in the presence of phenolphthalein until a light pink color appeared [16].

#### Determination of the Dry Matter

The determination of the dry substance was carried out using the refractometric method (SR 2213-5/2009). The product to be analyzed was diluted with distilled water in a specific proportion, and the refractive index of the resulting solution is determined with a refractometer (mention model number) [17].

#### Determination of Total Phenolic Content

The total polyphenol content (TPC) was quantified using a Shimadzu spectrophotometer with the Folin-Ciocalteu method. Sample preparation involved shredding and homogenization, followed by extraction with methanol. The extracts were concentrated and stored for analysis. The TPC of pine bud and pine bud fortified honey samples was determined following the methodology of Hussain et al. [14] with slight modifications. 0.5 mL of the tested samples was added to 2 mL of 0.5-N Folin-Ciocalteu reagent and mixed well. After 8 min, 4 mL of Na_2_CO_3_ was added while constantly stirring. The extracts were incubated for 2 h at 23 °C. The absorbance was measured at 760 nm using a UV -visible spectrophotometer (Hitachi − 2001, Hitachi Instruments Inc., Tokyo, Japan). A calibration curve of standard gallic acid was prepared. The total phenolic content was determined as GAE (gallic acid equivalents) in mg/100 g of pine or honey extracts.

#### DPPH Free-Radical-Scavenging-Assay

The DPPH radical scavenging activity of pine bud extracts and fortified honey was determined [17]. A DPPH. Solution was prepared by adding 2 mg of DPPH to 100 mL of 99.9% pure methanol. 50 µg/mL of the tested samples (pine bud and honey) was added to 50 µg/mL of the DPPH solution. The reaction mixture was incubated for 30 min in the dark. The absorbance was measured at a wavelength of 515 nm using a UV -visible spectrophotometer. Alpha-tocopherol was used as a positive control. DPPH solution in methanol served as the negative control. The scavenging percentage was calculated using the formula:

$$\:DPPH\:scavenging\:\%\:=\:A\left(negative\:control\right)\:-\:A\left(sample\right)\:/\:A\left(negative\:control\right)$$

#### FTIR Spectra

The pine bud extracts and fortified honey samples were subjected to FTIR analysis to identify the functional groups present in the 500–4000 cm^-1^ wavelength range. For this purpose, 0.01 g of each sample was homogenized with 0.01 g of anhydrous KBr using an agate mortar. A translucent pellet of the sample was then created by pressing at 1.2 Psi using a vacuum hydraulic press. The sample was exposed to an infrared beam and detected by a detector connected to a computer. The analysis results included the spectrum type, molecular binding form, specific functional groups, and chemical structure [18].

#### Statistical Analysis

Each experiment was conducted in triplicate and the results are presented as mean ± standard deviation. Tukey post-hoc test was conducted at a significance level of *p* < 0.05using IBM SPSS Statistics 2021. Pearson correlation analysis was performed using Microsoft Excel, and a heatmap was created to visualize the variations in phenolic compounds and maturation stages [19].

## Results and Discussion

### Humidity Level and Dry Matter Content

**Fig. 1:**
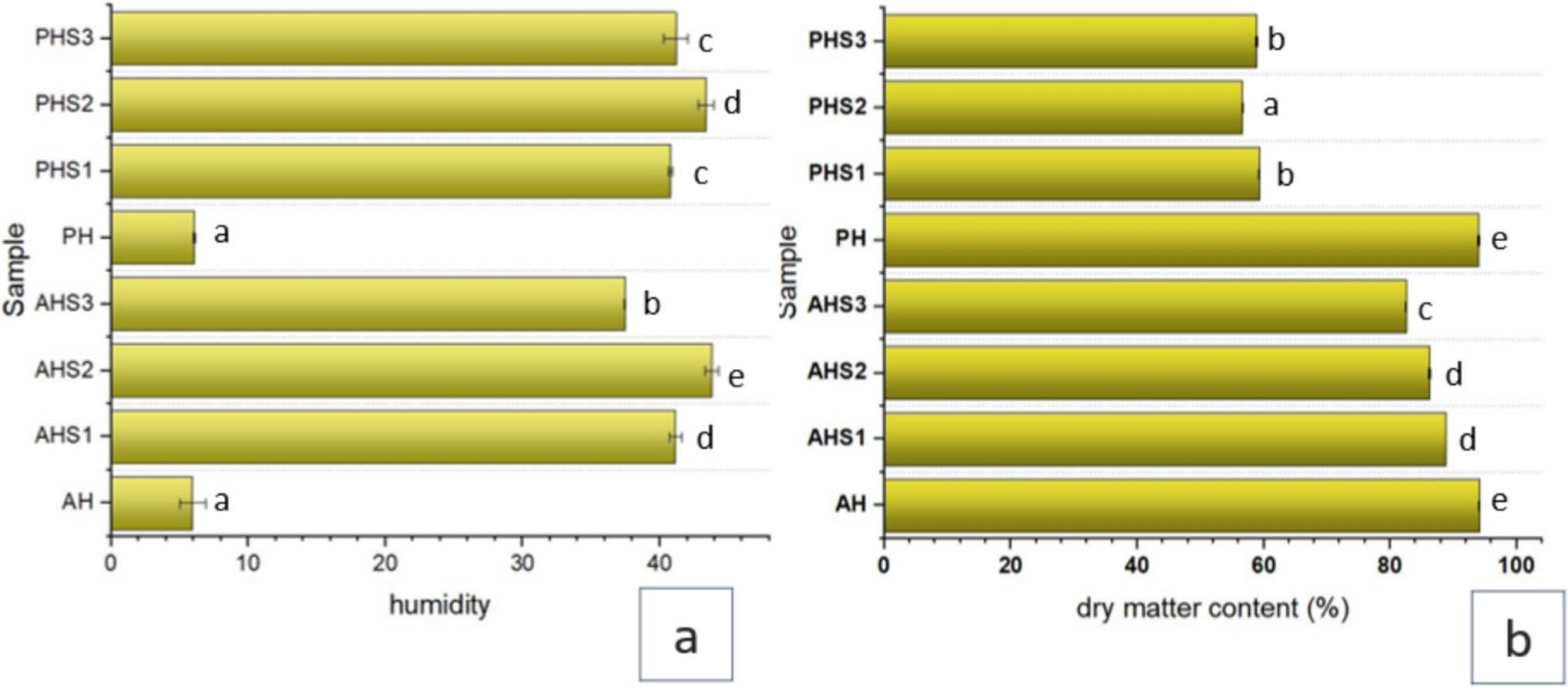
Humidity level and dry matter content (%) of Honey samples. Key: AH: Acacia honey, AHS1: Acacia honey fortified with maturation stage 1 pine bud extract, AHS2: Acacia honey fortified with maturation stage 2 pine bud extract, AHS3: Acacia honey fortified with maturation stage 3 pine bud extract, PH: Polyflower honey, PHS1: Polyflower honey fortified with maturation stage 1 pine bud extract, PHS2: Polyflower honey fortified with maturation stage 2 pine bud extract, PHS3: Polyflower honey fortified with maturation stage 3 pine bud extract. Letters on bars indicate significant differences among mean values. The letters on bars indicates the statistical differences among mean values analyzed using a t-Test (*p*< 0.05). The same letters on the bars indicate no significant difference, while different letters show significant differences among mean values

Moisture content is a crucial factor in determining the quality of honey, influenced by various factors such as beekeeping practices, honey maturity, and environmental conditions. Regulations stipulate that honey should not contain more than 20% moisture [20]. In our study, all tested samples met this requirement, with moisture levels below 20% (Fig. 1a). Acacia honey had a moisture content ranging from 1.18 to 8.2%, with the highest level observed in acacia honey biofortified with mature stage (S3) pine bud extract. On the other hand, polyflower honey showed higher moisture levels compared to acacia honey, with the highest level found in polyflower honey biofortified with S1 pine bud extract. Figure 1b illustrates that polyflower honey has a higher dry matter content (%) compared to acacia honey. Pine honey was observed to have a 17% moisture content [21]. Similarly, the moisture content of acacia honey was observed to be low in the study of Alaerjani and Mohammed [22]. Mellen et al. [23] observed a significant difference in dry matter content (%) of 15 types of samples originating from various natural sources.

### Total Phenolic Content and DPPH Free Radical Scavenging Percentage

**Fig. 2:**
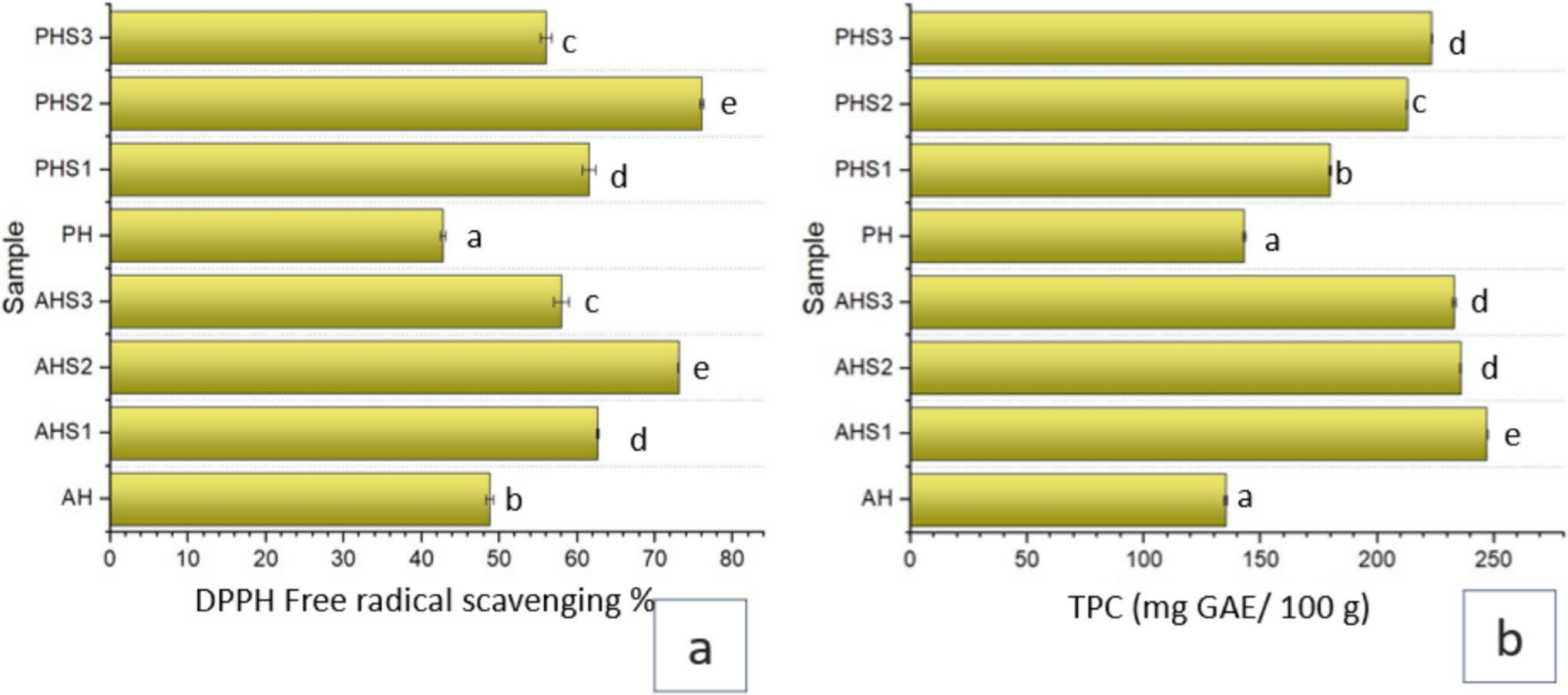
Antioxidant profile of Honey samples (**a**) total phenolic content (**b**) % DPPH free radical scavenging. Key: AH: Acacia honey, AHS1: Acacia honey fortified with maturation stage 1 pine bud extract, AHS2: Acacia honey fortified with maturation stage 2 pine bud extract, AHS3: Acacia honey fortified with maturation stage 3 pine bud extract, PH: Polyflower honey, PHS1: Polyflower honey fortified with maturation stage 1 pine bud extract, PHS2: Polyflower honey fortified with maturation stage 2 pine bud extract, PHS3: Polyflower honey fortified with maturation stage 3 pine bud extract. The letters on bars indicates the statistical differences among mean values analyzed using a t-Test (*p* < 0.05). The same letters on the bars indicate no significant difference, while different letters show significant differences among mean values

Some previous studies have documented a close association between total phenolic content and antioxidant capacity of honey which further depends on the botanical origin and concentration [24]. Biofortification had a significant impact on the total phenolic content (TPC) of both types of honey. Pure acacia honey and polyflower honey had TPC levels of 130 ± 0.5 and 175 mg GAE/ 100 g, respectively. These results are consistent with a previous study by Đurović et al. [25] which found that acacia honey had lower TPC content and was lighter in color. Starowicz et al. [26] documented that total phenolic content (TPC) and browning color of honey exhibited a significant positive association with each other. In the case of acacia honey biofortification, the highest TPC content was observed in AHS1 at 247 ± 0.04 mg GAE/ 100 g, while polyflower honey biofortified with pine bud extract at stage II of maturation exhibited a higher TPC content of 232.9 ± 0.9 mg GAE/100 g (Fig. 2a). The % DPPH scavenging activity of acacia honey was highest when biofortified with pine bud extract at maturation stage II (55 ± 0.05%), followed by SI biofortified honey at 52.64 ± 0.16%. Polyflower honey showed slightly lower antioxidant activity compared to acacia honey, with DPPH activity ranging from 51.5333 to 52.45% (Fig. 2b).

### Color Evaluation

The color of the honey samples was quantified during the study period. The results showed that throughout the biofortification process, acacia honey maintained a light color (L*) ranging from 35.44 ± 0.3^b^ to 38.59 ± 0.3^c^. Polyflower honey showed some variation in ‘L*’ values and was observed to darken during biofortification. Polyflower honey had higher a* values, particularly in PS2 and PS3, indicating a reddish color after biofortification. Acacia honey maintained a stable yellow hue (b* values) throughout biofortification, ranging from 12.54 ± 0.23^c^ in S1 to 13.53 ± 0.58^d^ in S3 (Table 1).

**Table 1:** Color evaluation of biofortified honey samples

Samples	L*	a*	b*
**Acacia honey**	35.44 ± 0.3^b^	11.59 ± 0.2^a^	8.99 ± 0.4^b^
**Acacia honey SI**	40.03 ± 0.32^e^	11.39 ± 0.12^a^	12.54 ± 0.23^c^
**Acacia honey S II**	38.59 ± 0.3^c^	12.86 ± 0.45^b^	13.53 ± 0.7^d^
**Acacia honey S III**	41.16 ± 0.33^f^	14.57 ± 0.5^c^	13.53 ± 0.58^d^
**Polyflower honey**	26.66 ± 0.9^a^	11.35 ± 0.8^a^	6.92 ± 0.8^a^
**Polyflower honey SI**	37.43 ± 0.3^c^	12.98 ± 0.3^b^	14.58 ± 0.35^e^
**Polyflower honey SII**	38.75 ± 0.5^d^	16.48 ± 0.3^d^	16.2 ± 0.9^f^
**Polyflower honey S III**	38.38 ± 0.76^d^	16.15 ± 0.78^d^	14.52 ± 0.79^e^

Key: L*, a* and b* are CIELAB color space, L* = lightness, a* and b* = unique colors

The same letters on the bars indicate no significant difference, while different letters show significant differences among mean values

### FTIR Analysis of Pine Bud Extracts and Fortified Honey

**Fig. 3:**
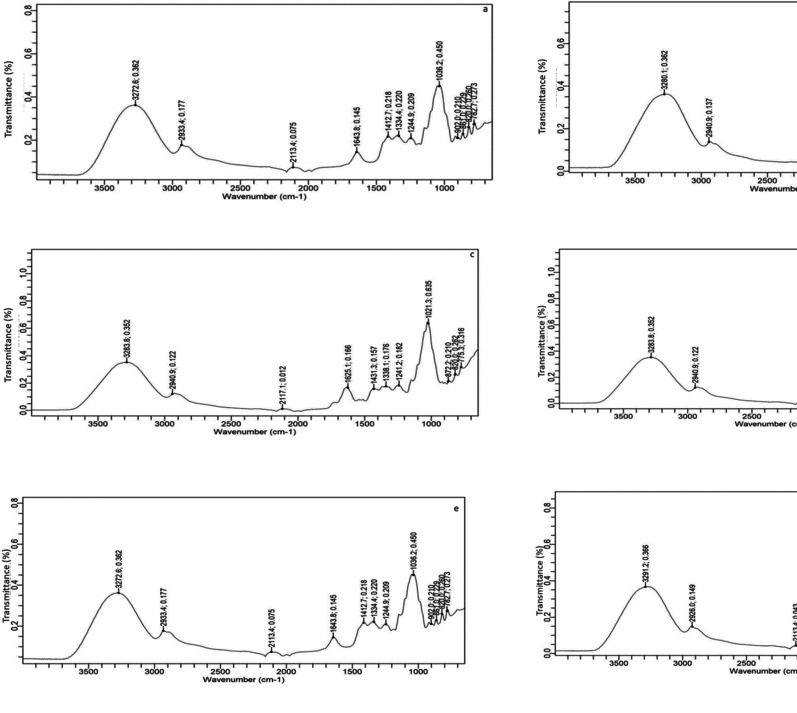
FTIR spectrum of honey biofortified with pine bud extracts (**a**) Acacia honey; **b** acacia honey foritified with pine bud extract during maturation stage I; **c** Acacia honey foritified with pine bud extract during maturation stage II; **d** Acacia honey foritified with pine bud extract during maturation stage III; **e** polyflower honey; **f** polyflower honey foritified with pine bud extract during maturation stage I; **g** polyflower honey fortified with pine bud extract during maturation stage II; **h** polyflower honey fortified with pine bud extract during maturation stage III

FTIR analysis revealed absorption patterns in both acacia and polyflower honey, indicating the presence of water, carbohydrates, and organic compounds typical of honey. Previous studies conducted by Anjos et al. [27] and Svečnjak et al. [28], have shown that the spectral area ranging from 3000 to 3500 cm^−1^ is attributed to O-H stretching in water, organic compounds, and sugars, while bands near 2900 cm^−1^ indicate C-H stretching in alkanes. A peak at 2900 cm⁻¹ corresponds to C-H stretching vibrations from aliphatic CH₂ and CH₃ groups, suggesting the presence of sugars. A peak around 1700–1750 cm⁻¹ in both samples represents C = O stretching vibrations from carbonyl groups, commonly found in honey due to its organic acid content. Peaks at 1600–1650 cm⁻¹ indicate C = C stretching vibrations from aromatic or unsaturated compounds. The region between 1400 and 1500 cm⁻¹ shows bending vibrations from C-H and O-H groups, confirming the presence of sugars. A cluster of peaks between 1000 and 1200 cm⁻¹, known as the fingerprint region, reveals C-O stretching vibrations from alcohols, ethers, and carbohydrates, indicating the complex sugar composition in both acacia and polyflower honey (Fig. 3e and h). Kozłowicz et al. [29] documented that the chemical structure of carbohydrates exhibited stretching vibrations of C-C, C-H, and C-O, and bending vibrations of C-H.

### Pearson Correlation

**Fig. 4:**
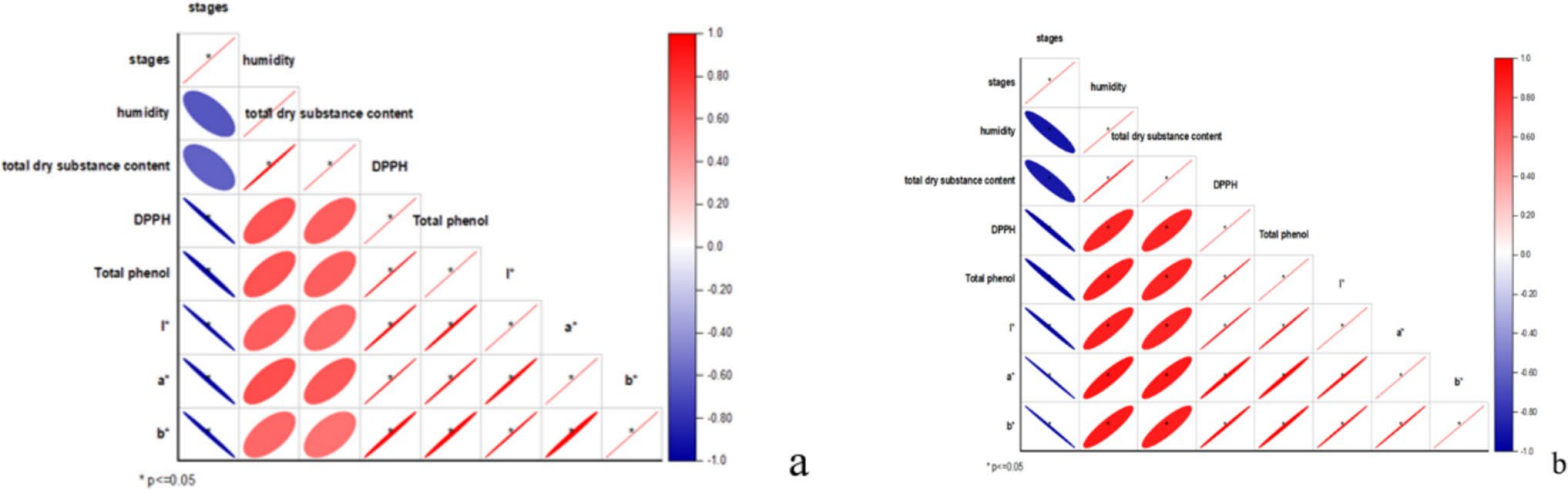
Correlation analysis examines the relationship between the physiochemical properties of both Acacia honey (**a**) and polyflower honey (**b**) biofortified with pine bud extracts at different maturation stages. Statistical analysis was conducted using a t-test (*p* < 0.05)

Pearson correlation analysis was conducted to examine the relationship between various factors in two types of honey (Fig. 4a and b). In polyflower honey, a strong positive correlation was found between dry substance matter and humidity (r^2^ = 0.98). DPPH scavenging percentage and total phenolic content showed a positive correlation, indicating the importance of phenolic content in enhancing antioxidant properties. The stages of honey had a negative correlation with color attributes, particularly lightness (L*). In acacia honey, humidity was observed to have a strong positive correlation with total dry substance (r^2^ = 0.997) and DPPH (r^2^ = 0.995). DPPH exhibited strong positive correlations with total phenolic content (0.99064) and color attributes L* and a* (r^2^ = 0.985; r^2^ = 0.98) respectively. Starowicz et al. [26] documented that total phenolic content (TPC) and browning color of honey exhibited a significant positive association with each other. Correlations between total phenol concentration and antioxidant activity were studied in honeys from Poland, Italy, and Romania. Studies by Kus et al. [30], Rosa et al. [31], and Al et al. [32] confirmed this relationship. Similarly, in Serbian honey, Gorjanović et al. [33] and Savatović et al. [34] observed a strong positive correlation between total phenol content and antioxidant potential using DPPH techniques. Milivojević et al. [35] and Đurović et al. [25] also reported a significant positive correlation between antioxidant activity and phenol content.

## Conclusion

The results indicated that the biofortification of Acacia and polyflower honey with pine bud extracts at different maturation stages significantly influence the moisture content, dry matter content, antioxidants, total phenolic content, and color. Both types of honey maintained a standard humidity level of less than 20% throughout the biofortification process. The antioxidant profile of Acacia honey, measured by DPPH and TPC content, showed a slight increase as the biofortification progressed. Acacia honey fortification showed highest TPC content, *i.e*., 247 ± 0.04 mg GAE/ 100 g at SI, while polyflower honey 232.9 ± 0.9 mg GAE/ 100 g at SII. The DPPH free radical scavenging activity of acacia honey was highest 55 ± 0.05% at SII, Color analysis indicated a slight change in lightness L*, a* and b* values during the biofortification process. Polyflower honey exhibited more pronounced color changes compared to Acacia honey throughout the biofortification process. These results suggest that pine bud extracts enhance honey’s nutritional and functional properties, with potential applications in food and nutraceutical industries. Further research is needed to grow the applications of this biofortification technique.

## Data Availability

No datasets were generated or analysed during the current study.
